# The detection of long‐lasting memory foot‐and‐mouth disease (FMD) virus serotype O‐specific CD4^+^ T cells from FMD‐vaccinated cattle by bovine major histocompatibility complex class II tetramer

**DOI:** 10.1111/imm.13367

**Published:** 2021-06-08

**Authors:** Shuya Mitoma, Brigid Veronica Carr, Yongjie Harvey, Katy Moffat, Satoshi Sekiguchi, Bryan Charleston, Junzo Norimine, Julian Seago

**Affiliations:** ^1^ Department of Veterinary Medicine Faculty of Agriculture University of Miyazaki Miyazaki Japan; ^2^ The Pirbright Institute Woking Surrey UK

**Keywords:** foot‐and‐mouth disease vaccine, memory T cell, tetramer

## Abstract

Foot‐and‐mouth disease (FMD) is a highly contagious, economically devastating disease of cloven‐hooved animals. The development of long‐lasting effective FMD vaccines would greatly benefit the global FMD control programme. Deep analysis of adaptive immunity in cattle vaccinated against FMD is technically challenging due to the lack of species‐specific tools. In this study, we aimed to identify CD4^+^ T‐cell epitopes in the FMD virus (FMDV) capsid and to phenotype the CD4^+^ T cells that recognize them using bovine major histocompatibility complex (BoLA) class II tetramer. A BoLA class II tetramer based on the *DRA*/DRB3*020:*02* allele and FMDV antigen‐stimulated PBMCs from bovine vaccinates were used to successfully identify four epitopes in the FMDV capsid, three of which have not been previously reported; two epitopes were identified in the structural protein VP1, one in VP3 and one in VP4. Specificity of the three novel epitopes was confirmed by proliferation assay. All epitope‐expanded T‐cell populations produced IFN‐γ *in vitro*, indicating a long‐lasting Th1 cell phenotype after FMD vaccination. VP3‐specific CD4^+^ T cells exhibited the highest frequency amongst the identified epitopes, comprising >0·004% of the CD4^+^ T‐cell population. CD45RO^+^CCR7^+^ defined central memory CD4^+^ T‐cell subpopulations were present in higher frequency in FMDV‐specific CD4^+^ T‐cell populations from FMD‐vaccinated cattle *ex vivo*. This indicates an important role in maintaining cell adaptive immunity after FMD vaccination. Notably, FMDV epitope‐loaded tetramers detected the presence of FMDV‐specific CD4^+^ T cells in bovine PBMC more than four years after vaccination. This work contributes to our understanding of vaccine efficacy.

## Graphical abstract

We have used bovine MHC II tetramers and PBMC from cattle vaccinated against foot‐and‐mouth disease (FMD) to identify novel CD4+ T‐cell epitopes in the capsid of FMD virus (FMDV) and to confirm the presence of long‐lasting FMDV‐specific CD4+ T cells. Ex vivo analyses revealed the presence of CD45RO+CCR7+ central memory cells in higher frequency and epitope‐expanded T‐cell populations produced IFN‐γ in vitro indicating a Th1 cell phenotype. This work indicates an important role in maintaining cell adaptive immunity after FMD vaccination and contributes to our understanding of vaccine efficacy.

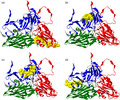

**Abbreviations**
FMD: Foot‐and‐mouth disease
FMDV: foot‐and‐mouth disease virus
SAT: Southern African Territories
CTL: cytotoxic T lymphocytes
CD: cluster of differentiation
TCR: T‐cell receptor
MHC: major histocompatibility complex
BoLA: bovine leucocyte antigen
TGEM: tetramer‐guided epitope mapping
PBMCs: peripheral blood mononuclear cells
BEI: binary ethylenimine
FMD O1M: FMDV‐O1/Manisa/Turkey/69
FBS: fetal bovine serum
CCR7: C‐C motif chemokine receptor 7
CFSE: carboxyfluorescein succinimidyl ester

## INTRODUCTION

Foot‐and‐mouth disease (FMD) is an economically devastating and highly contagious animal disease that infects cloven‐hoofed animals, including domesticated cattle, sheep, goats and pigs. Its causative agent, FMD virus (FMDV), belongs to the *Picornaviridae* family, *Aphthovirus* genus, and is categorized to seven major serotypes, termed O, A, C, Asia1, Southern African Territories (SAT)1, SAT2 and SAT3. Current FMD vaccines are comprised of inactivated virus preparations and must be derived from antigenically similar strains within the same serotype to confer effective protection [1]. Vaccine efficacy is positively correlated with antigen integrity, and it is important that the capsid of inactivated virions, comprised of the structural proteins VP1, VP2, VP3 and VP4, is intact [2]. In FMD‐endemic areas, regular vaccination is required to maintain protective immunity against FMDV [3]. Such regular vaccination is a burden for farmers in terms of cost and workload. To develop long‐lasting effective FMD vaccines, it is important to understand how immunological memory and protective immunity is established following vaccination.

During a protective immune response against virus infection, CD4^+^ T cells work as helper T cells mainly for maturing B cells and activating cytotoxic T lymphocytes (CTL). In cattle vaccinated against FMD, the presence of circulating memory CD8^+^ T cells [4] and T‐cell‐dependent memory B‐cell responses have been confirmed by *in vitro* assay [5]. Both of these cell types contribute to FMDV clearance and could be elicited by helper CD4^+^ T cells. In FMD‐vaccinated cattle, helper T cells play an important role in influencing the major proliferating phenotype and IFN‐γ production of effector cells [6], as well as mediating the production of antibodies and class switching [7]. To date, it has been difficult to verify FMD vaccine efficacy over long periods of time, specifically that mediated by CD4^+^ T cells, without viral challenge because the frequency of memory cells is lower than the detectable limit using conventional immunological methods. However, major histocompatibility complex (MHC) class II tetramer‐based methods offer the required analytical depth and the opportunity to better characterize such memory CD4^+^ T cells. In this study, MHC class II tetramer was employed as a labelling tool to detect antigen‐specific CD4^+^ T cells using the binding property of peptide/MHC class II molecule complex to a specific T‐cell receptor (TCR). Tetramer assays are used for single‐cell phenotyping and enumeration and have the advantage that they enable the recovery and further study of sorted cells based on tetramer staining (Nepom *et al*., 2012) [8].

Importantly, in order to detect and analyse the memory CD4^+^ T cells using bovine leucocyte antigen (BoLA) class II tetramers, their respective epitopes must first be identified. To date, limited success has been achieved in the identification of CD4^+^ T‐cell epitopes within the FMDV structural proteins, and the respective epitope and MHC class II combination has been estimated [9, 10, 11, 12, 13, 14, 15]. With regard to the direct identification of epitope–MHC class II combinations [16], it was shown that mouse L cells exogenously expressing the BoLA‐DRA/DRB3*001:01 molecule were able to present the FMDV15 peptide [17] to bovine CD4^+^ T cells. In 2005, Norimine *et al*. [18] used artificial antigen‐presenting cells transfected with a single BoLA class II molecule and CD80/CD86 to demonstrate that *Anaplasma Marginale* CD4^+^ T‐cell epitopes are restricted by certain BoLA class II (DR and DQ). Although these studies were able to investigate T‐cell proliferative responses to specific epitopes and identified restricted BoLA class II alleles, all possible combinations of alpha and beta chains needed to be examined, and therefore, this approach has not been commonly used. Human MHC class II tetramers have also been used to facilitate the analysis of antigen‐specific CD4^+^ T cells and to perform tetramer‐guided epitope mapping (TGEM) [19, 20, 21].

Here, we show that FMDV‐specific memory CD4^+^ T cells can be detected long after FMDV vaccination using the TGEM method and show they represent long‐lasting CD4^+^ T‐cell responses against epitopes within the structural proteins of FMDV.

## MATERIALS AND METHODS

### Animals and their haplotypes

The two animals used in this study, named FMD7 and FMD9 and described elsewhere [4, 7, 22], were bovine MHC serotype A18 heterozygotes, which had been vaccinated twice with a commercial oil‐adjuvanted monovalent O1/Manisa FMD (accession number: AAT01766·1) vaccine (Merial Animal Health Ltd); a prime and boost regimen was used, with the boost administered 11 weeks after prime vaccination. The bovine peripheral blood mononuclear cells (PBMCs) used for this study were collected over 4 years (55‐56 months) after the boost vaccination and cryopreserved in liquid nitrogen. All animal experiments were carried out with the approval of the local Ethics Committee at the Pirbright Institute, and in accordance with the Home Office Guidance on the Operation of the Animals (Scientific Procedures) Act 1986 and associated guidelines. Animal haplotypes were determined using RNA extracted with TRIzol reagent (Invitrogen) and DNA extracted using the NucleoSpin Blood Mini Kit (Macherey‐Nagel, Düren, Germany). BoLA‐DRB3 exon2 RFLP‐PCR methods [23] and DNA sequencing identified the BoLA MHC II serotype A18‐related *BoLA*‐DRB3*020:*02* allele. The complete mRNA coding sequence of *BoLA*‐DRB3*020:*02* (accession number: MW052496·1) for construction of the BoLA class II tetramer was determined by sequencing cDNA generated from *BoLA*‐*DRB3* mRNA from A18 homozygous cattle using followed primers, DRB3F: 5’‐GGCATGGTGTGCCTGTATTTCTCTG‐3’ and DRB3R:5’‐TCAGCTCAGGAGCCCTGTTGG‐3’.

### Antigen

Intact FMDV‐O1/Manisa/Turkey/69 (FMD O1 M) antigen was used to stimulate the FMDV‐specific cells in PBMCs. The antigen was prepared by binary ethylenimine (BEI) inactivation of virus and subsequent sucrose density gradient purification as previously described [2]. Antigen was stored in single‐use aliquots at −80℃ at a concentration of 157 µg/ml, and thawed aliquots were assessed for capsid integrity by ThermoFluor assay as reported [24].

### Peptides

As previously described [10] and [11], an overlapping peptide library representing the four structural capsid (VP1‐VP4) proteins of FMDV‐O/UKG/35/2001 [25] was used. The peptide library was comprised of 137 peptides,each peptide was 15 amino acids in length and overlapped the following peptide by 10 amino acids. For initial screening of epitope mapping, 27 pools were prepared with each pool containing four to six peptides.

### Construction of soluble BoLA class II expression vector

A single cassette encoding soluble BoLA class II dimer, based on *DRA* (accession number: NM_001012677·1) and DRB3*020:*02* (accession number: MW052496·1), was inserted into the pcDNA3·1 expression plasmid to generate p2ADR*020:02/pcDNA3·1 (Figure S1) [26]. The cassette contained a furin cleavage site and the FMDV‐derived 2A peptide sequence [27] and [28] between BoLA class II alpha and beta chains to facilitate chain separation. Soluble BoLA class II dimer was prepared by transfecting pcDNA3·1/p2ADR*020:02 into Expi293 cells (Gibco) and purification using anti‐FLAG M2 affinity gels (Sigma‐Aldrich). The purified product was biotinylated with BirA Biotin‐Protein Ligase Kit (Avidity, Denver, CO, USA).

### Construction of BoLA class II tetramer

Biotinylated soluble BoLA class II molecules (0·5 mg/mL) were loaded with peptides by incubation with fivefold molar excess of individual peptide or ~20‐ to 30‐fold molar excess peptide pools (each containing 4 ~ 6 peptides) for 48 hours at 37℃ in 100 mM sodium phosphate, pH 6·0, and 0·2% n‐octyl‐D‐beta‐glycosidase (Sigma‐Aldrich). To construct the BoLA class II tetramer complex, phycoerythrin‐conjugated streptavidin (PE‐SA) (eBioscience, Carlsbad, CA, USA) was added stepwise to a final molar ratio of 8 MHC class II complexes to 1 PE‐SA by incubating for 15 min between additions at room temperature.

### PBMC restimulation and epitope mapping using BoLA class II tetramer

PBMCs were maintained and propagated in RPMI‐1640 medium, with GlutaMAX^TM^ supplement, HEPES (Gibco) containing 10% fetal bovine serum (FBS), 50 µM 2‐mercaptoethanol (Gibco) and 100 IU/mL penicillin, 100 µg/mL streptomycin (Gibco), herein described as complete RPMI medium (cRPMI). PBMCs, plated at a density of 2·5 × 10^6^ cells/ml in 48‐well plates, were stimulated by incubating with the BEI FMDV‐O1 M intact antigen at 0·1 µg/mL for six days at 37℃, 5% CO_2_. After six‐day culture, the PBMCs were transferred to new wells and treated with fresh cRPMI medium and 0·5 ng/ml of recombinant bovine interleukin‐2 (rboIL‐2, [29] every other day until flow cytometry analysis on days 12‐16. Prior to adding fresh medium and rboIL‐2, 500 µL of culture supernatant was gently removed from the surface without disturbing the cells. When cells reached confluency, they were split and transferred to new wells. To stain the cultured cells, 250 µl aliquots of cell suspension were transferred to wells of a 96‐well plate. After centrifugation at 300 × g for 5 min and removal of the supernatant, the cells were stained by incubation with 10 µg/mL of each pooled or individual peptide‐loaded tetramer–PE in cRPMI for 1 hour at 37℃. The tetramer‐stained cells were also stained with 1 µg/mL of anti‐bovine CD4‐FITC antibody (CC8, IgG2a, Bio‐Rad) in cRPMI for 10 min at room temperature and then with 2·5 µg/mL of the viability stain 7‐aminoactinomycin D (7‐AAD; Invitrogen) /PBS for 10 min at room temperature. Table 1 shows the antibodies and viability fluorescents used in this study. The cells were fixed with 1% paraformaldehyde (PFA) /PBS solution after washing twice with PBS, and the following day, data were acquired with DIVA8 software using a BD LSRFortessa™ flow cytometer and then analysed in FCS Express 6·0 (Pasadena, CA, U.S.A., De Novo Software). The location of identified DRA/DRB3*020:02‐restricted FMDV epitopes was analysed using SMTL ID: 5nej.1, CryoEM structure of FMDV‐O1/Manisa/Turkey/69 models [30] on the Vector NTI Express Designer software (Life Technologies).

**TABLE 1:** Antibody or staining reagents used in this study

Antibody /cell staining reagent	Clone, Isotype	Label‐fluorescent
Mouse anti‐bovine CD4 (Bio‐Rad)	CC8, IgG2a	FITC (Bio‐Rad) or Alexa 647 (Bio‐Rad)
Mouse anti‐bovine CD8 (Bio‐Rad)	CC63, IgG2a	no‐label or APC‐Cy7, lightning‐link (abcam)
Mouse anti‐bovine TCR1‐N24 delta chain (Washington State University)	GB21A, IgG2b	no‐label or APC‐Cy7, lightning‐link (abcam)
Mouse anti‐bovine CD14 (Bio‐Rad)	CC‐G33, IgG1	no‐label or APC‐Cy7, lightning‐link (abcam)
Mouse anti‐bovine CD21 (Bio‐Rad)	CC21, IgG1	no‐label or APC‐Cy7, lightning‐link (abcam)
Mouse anti‐bovine IFN‐γ (Bio‐Rad)	CC302, IgG1	FITC (Bio‐Rad)
Mouse anti‐bovine IL−4 (Bio‐Rad)	CC303, IgG2a	FITC (Bio‐Rad)
Mouse anti‐human IL−17A (Mabtech)	MT504, IgG1	FITC (Mabtech)
Mouse anti‐bovine CD45RO (Bio‐Rad)	IL‐A116, IgG3	PerCP/Cy5·5, lightning‐link (abcam)
Rat anti‐human CCR7 (BD Pharmingen)	3D12, IgG2aκ	Alexa 647(BD Pharmingen)
Mouse IgG1 isotype control (Bio‐Rad)	^a^Cat:MCA928F, IgG1	FITC (Bio‐Rad)
Mouse IgG2a isotype control (Bio‐Rad)	OX−34, IgG2a	FITC (Bio‐Rad)
Mouse IgG3 isotype control (Bio‐Rad)	^a^Cat:MCA5920, IgG3	PerCP/Cy5·5, lightning‐link (abcam)
Rat IgG2aκ isotype control (BD Pharmingen)	R35‐95, IgG2aκ	Alexa 647(BD Pharmingen)
CellTrace CFSE cell proliferation kit (Invitrogen)		
7‐Aminoactinomycin D (7‐AAD, Invitrogen)		
Fixable Viability Dye eFluor 780 (Invitrogen)		

^a^ Catalogue number of mAb.

### Cell proliferation assay

To confirm the specificity of tetramer staining, cell proliferation assays were conducted using three peptides, p220 (VP3), p248 (VP1) and p432 (VP4), identified by TGEM. PBMCs were thawed and transferred (3 × 10^6^/well) to a 48‐well plate prior to incubation for 1 hour at 37℃. After incubation, non‐adherent cells were collected and depleted of CD8‐, γδ‐, CD14‐, CD21‐positive cells using a cocktail of Abs (anti‐CD8; CC63, Bio‐Rad, anti‐TCR1‐N24 delta chain; GB21A, Washington State University, anti‐CD14; CC‐G33, Bio‐Rad, anti‐CD21; CC21, Bio‐Rad; Table 1) and MACS anti‐mouse IgG microbeads (Miltenyi Biotec, Auburn, CA, USA). Following three independent enrichments, CD4^+^ cells comprised 40‐55% of the collected lymphocyte population, compared with 20‐25% prior to enrichment. After washing the CD4^+^‐enriched cells twice with PBS, they were stained with 1 µM CellTrace CFSE (Invitrogen) for 10 min at 37℃. Stained cells were then washed twice with cold cRPMI medium, and subsequently added back at a density of 1·5 × 10^6^ cells/well to the wells with adherent cells. Antigen stimulation was conducted in the presence of 10 µg/ml individual peptide or 0·1 µg/mL BEI FMDV‐O1 M intact antigen overnight at 37℃, and the culture medium was replenished with 1 ml/well the next day. After 6‐day culture, the cells were stained with peptide‐loaded tetramer–PE for 1 hour at room temperature and then with anti‐bovine CD4–Alexa 647 antibody (CC8, IgG2a, Bio‐Rad) for 20 min at room temperature. Stained cells were fixed with 1% PFA/PBS and analysed using flow cytometry within 24 hours.

### Expansion of peptide‐specific T cells

To expand peptide‐specific CD4^+^ T cells for further analysis, CD4^+^‐enriched PBMCs in the presence of autologous adherent cells were stimulated with identified peptide and then expanded with rboIL‐2 over a total period of four weeks. CD4^+^ T‐cell‐enriched bovine PBMCs were transferred to a 48‐well plate at a density of 1·5 × 10^6^ cells/well and then stimulated with 10 µg/mL of individual peptide in cRPMI medium as described for cell proliferation assays. After 6 days of stimulation, cells were transferred to new wells and expanded with 0·5 ng/mL rboIL‐2 until day 14 as described for PBMC restimulation. On day 14, expanded CD4^+^ T cells were stimulated again with individual peptide and autologous adherent cells for 6 days and expanded with 0·5 ng/mL rboIL‐2 until further analysis.

### Intracellular cytokine staining to determine FMDV‐specific CD4^+^ T‐cell subsets

Intracellular cytokine staining was performed using 4‐week expanded peptide‐specific T cells. Cell mixtures, each consisting of 1 × 10^6^ expanded peptide‐specific cells and 1 × 10^6^ autologous PBMCs, were cultured with 5 µg/mL of the respective peptide or medium only in a 48‐well plate overnight at 37℃ with CO_2_. After peptide stimulation, 5 µg/mL of brefeldin A (Bio‐Rad) was added to the cells, followed by incubation for 4 hours at 37℃. Stimulated cells were collected and stained with 5 µg/mL of BoLA class II tetramer–PE in cRPMI for 1 hour at room temperature and then with anti‐bovine CD4‐Alexa 647 mAb in cRPMI for 20 min at room temperature. Stained cells were fixed and permeabilized using Leucoperm™ (Bio‐Rad) after washing twice with 2% FBS/PBS. Permeabilized cells were immediately stained with 1 µg/mL of mouse anti‐bovine IFN‐γ‐FITC (cc302, IgG1, Bio‐Rad), anti‐bovine IL‐4 FITC (CC303, IgG2a, Bio‐Rad), anti‐human IL‐17A FITC (MT504, IgG1, Mabtech) or mouse isotype control antibody‐FITC (IgG1 and IgG2a, Bio‐Rad) in cRPMI for 20 min at room temperature. After washing cells twice, cells were suspended in 1% PFA/PBS and immediately analysed by flow cytometry.

### Staining *ex vivo* PBMCs to determine the memory phenotype and frequency of FMDV‐specific CD4^+^ T cells

To determine the phenotype of FMDV‐specific CD4^+^ T cells still present after vaccination, the cryopreserved PBMCs were stained with tetramers and antibodies that recognize memory phenotype markers. After thawing of the PBMCs, cells were cleaned up with Histopaque 1083 (Sigma‐Aldrich) and passed through a 40‐µm cell strainer (Corning, Glendale, Arizona, USA). The thawed PBMCs were stained in cRPMI with 5 µg/mL of individual peptide‐loaded BoLA class II tetramer–PE for 1 hour at room temperature and then stained with anti‐bovine CD4‐FITC, CD45RO‐PerCP/Cy5·5, anti‐human CCR7‐Alexa 647 antibodies and the cocktail antibodies (anti‐CD8, anti‐TCR1‐N24 delta chain, anti‐CD14 and anti‐CD21) conjugated with APC‐Cy7 (lightning‐link kit, abcam); staining was performed by Ab addition for the last 15 min to the tetramer staining cells. A separate aliquot of the same PBMC was stained with mouse IgG3‐PerCP/Cy5·5 and rat IgG2a κ‐Alexa 647 isotype control antibodies to decide the quantile line. After staining with antibodies, cells were washed twice with 2% FBS/PBS and stained with fixable viability dye eFluor 780 (eBioscience). To calculate the frequency of peptide‐specific CD4^+^ T cells, the stained cells were divided into two groups. Ten per cent of the stained cells were analysed to calculate the CD4^+^ T‐cell population in PBMCs and 90% to calculate the CD4^+^ tetramer^+^ population in PBMCs after tetramer^+^ cells had been positively selected using a PE microbead enrichment protocol as previously described [31, 32]. CD4^+^ T cells, *ex vivo*, were analysed after gating on the lymphocyte region, excluding dead and CD8^+^, γδ^+^, CD14^+^, and CD21^+^ cells.

## RESULTS

### Identification of novel CD4^+^ T‐cell‐restricted epitopes in the FMDV capsid using tetramer‐guided epitope mapping (TGEM)

**FIGURE 1:**
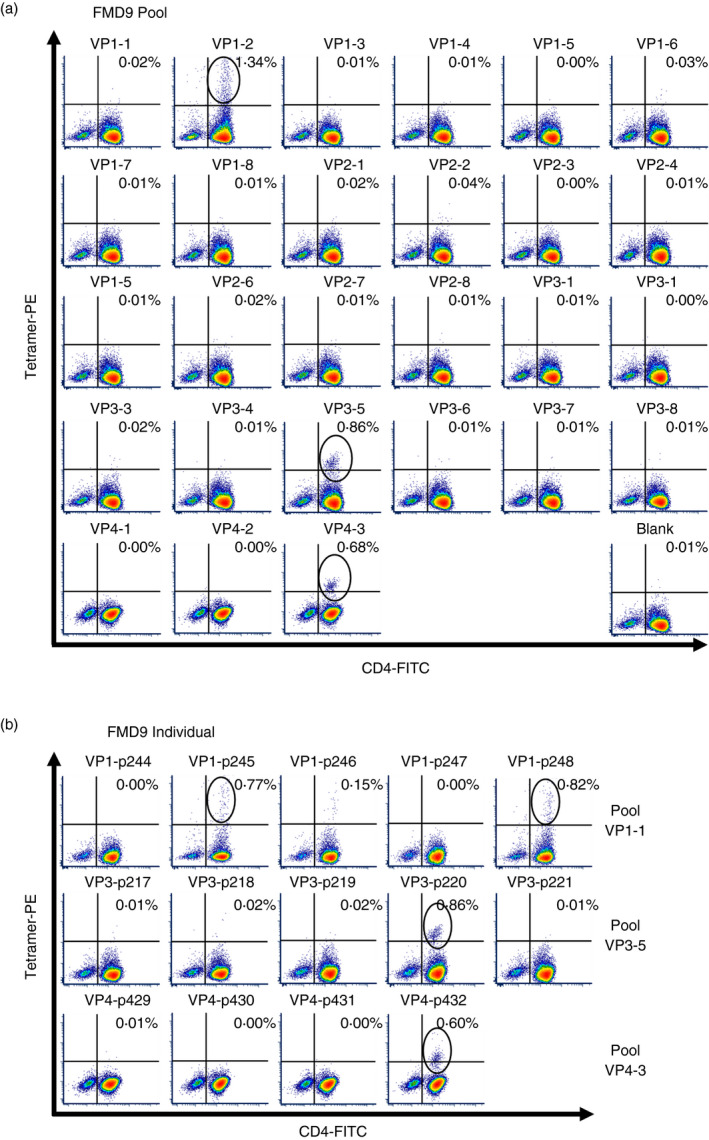
Identification of DRA/DRB3*020:02 molecule‐restricted CD4^+^ T‐cell epitopes within the FMDV capsid. PBMCs from animal FMD9, heterozygous for the MHC II DRB3*020:*02* allele, were stimulated with inactivated, intact FMDV (strain O1/Manisa) antigen for 6 days and then incubated for a further 6 to 10 days in the presence of rboIL‐2. PBMCs were then stained with pooled (a) or individual (b) peptide‐loaded tetramers and analysed by flow cytometry. Cells were gated on lymphocytes, singlets and then live cells. Black circles indicate CD4^+^ tetramer^+^ cell populations, confirming the recognition of a FMDV capsid epitope–tetramer complex by CD4^+^ T cells. Data sets for VP1 to VP3, and for VP4, FMDV structural proteins were acquired on two separate days. These data are representative of two independent experiments using two animals

**FIGURE 2:**
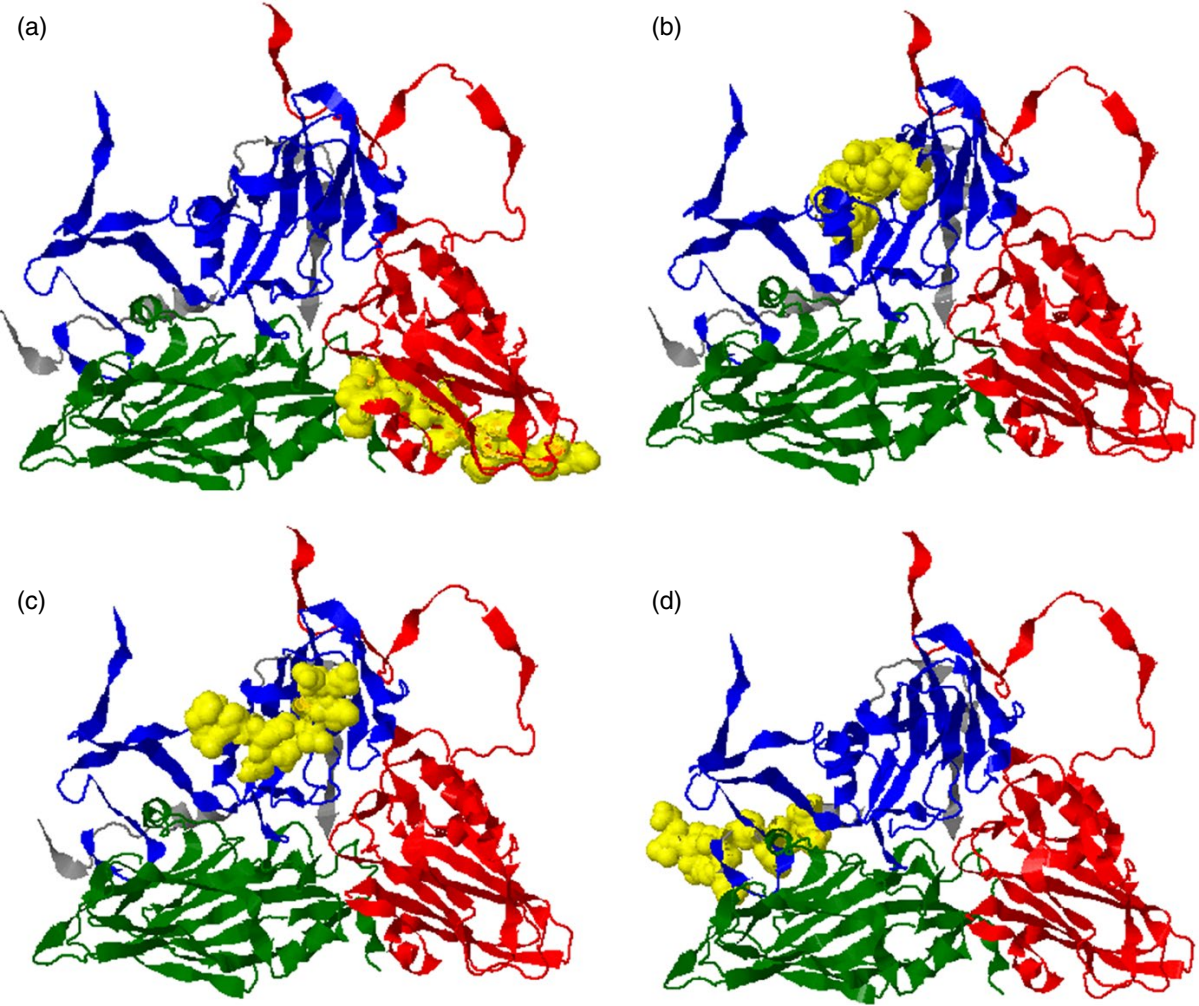
Position of identified epitopes on the protomer of FMDV capsid. Structures (a‐d) are based on the protein data bank: 5nej.1, FMDV‐O1/Manisa model [30] and depict the externally viewed capsid. The structural proteins VP1, VP2, VP3 and VP4 are shown in blue, green, red and grey, respectively. Yellow surface regions indicate the locations of identified epitopes. (a) p220 (VP3: 116‐130), (b) p245 (VP1: 32‐45), (c) p248 (VP1: 46‐60) and (d) p432 (VP4:71‐85)

To identify CD4^+^ T‐cell‐restricted epitopes in the FMDV capsid, we performed epitope mapping using peptide‐loaded BoLA class II tetramers to screen antigen‐stimulated PBMC derived from MHC II‐matched vaccinated cattle (termed FMD7 and FMD9). Initial screening was conducted using DR*020:02 tetramer loaded with peptide pools, which each comprised of 4‐6 peptides and corresponded to a region of the FMDV (strain O/UKG/35/2001) VP1‐4 capsid proteins. The external VP1‐3 capsid proteins were each represented by 8 peptide pools, and the comparatively smaller, internal VP4 capsid protein by 3 pools. PBMCs were stimulated with BEI‐inactivated FMDV‐O1/Manisa, the capsid of which shares 97·8% amino acid sequence homology with O/UKG/35. TGEM analyses using antigen‐stimulated PBMC, derived from both animals FMD7 and FMD9, showed pooled peptide‐loaded DR*020:02 tetramer‐positive cell populations with pools VP1‐2 and VP3‐5 (Figure 1a, Figure S2a). An additional tetramer‐positive cell population was identified using animal FMD9 PBMC and pool VP4‐3 (Figure 1a). After initial epitope mapping screens, expanded cells were stained with individual peptide‐loaded DR*020:02 tetramer. Subsequent TGEM analyses using antigen‐stimulated FMD9 PBMC and individual peptide‐loaded tetramer revealed the DRA/DRB3*020:02 molecule‐restricted FMDV epitopes as p245 (VP1: 31‐45), p248 (VP1: 46‐60), p220 (VP3: 116‐130) and p432 (VP4:71‐85) (Figure 1b, Table 2), while similar analyses using FMD7 PBMC showed the epitopes as p248 (VP1) and p220 (VP3) (Figure S2b, Table 2). However, the p245‐restricted MHC class II tetramer was only detected once as a positive population above the threshold (0·5%) using FMD9 PBMC in two independent experiments. Structural analysis of the locations of p220 and p245 in the VP1 and VP3 proteins, respectively, suggests that they are not exposed to either the internal or external capsid surfaces (Figure 2a,b). In contrast, both terminals of p248 appear to be exposed on the external surface of the VP1 capsid protein, while the middle portion appears hidden (Figure 2c). Peptide 432 is located at the C‐terminal of the VP4 internal capsid protein (Figure 2d).

**TABLE 2:** Identified peptides showing DR*020:02 tetramer‐positive staining

Name	Position	Sequence	FMD7	FMD9
p220	VP3: 116‐130	DAKARYMIAYAPPGM	○	○
p245	VP1: 31‐45	DVSFILDRFVKVTPK		○
p248	VP1: 46‐60	DQINVLDLMQTPAHT	○	○
p432	VP4: 71‐85	LASSAFSGLFGALLA		○

### FMDV capsid epitopes induce specific CD4^+^ T‐cell proliferation

**FIGURE 3:**
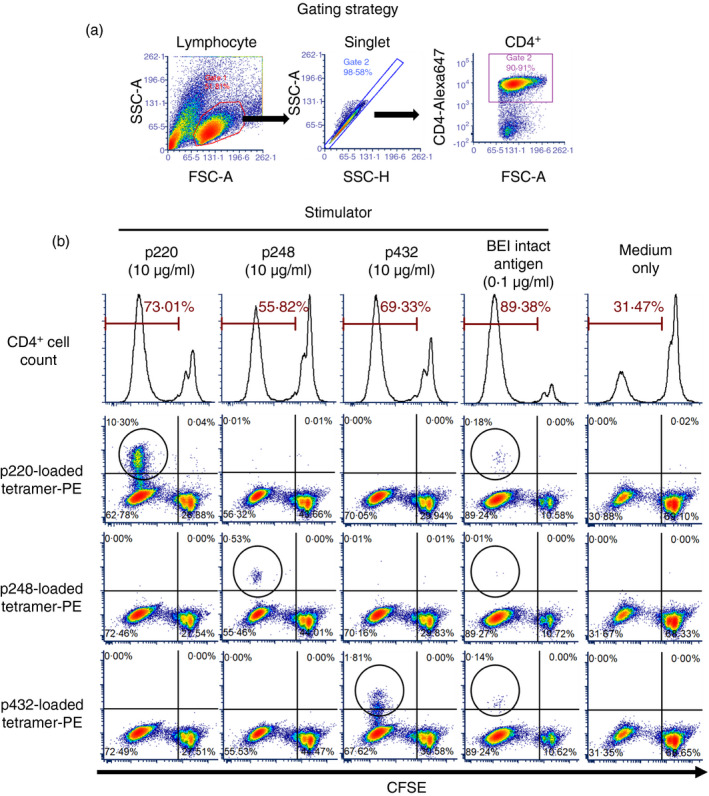
Tetramer staining was specific and correlated with CFSE proliferation results. CD4^+^ T‐cell‐enriched cell populations were stained with CFSE, then stimulated with individual peptide (p220, p248 or p432) or intact, inactivated FMDV and cultured for 6 days. After staining with individual peptide‐loaded tetramer–PE and anti‐CD4‐Alexa 647, data were acquired by flow cytometry. (a) Dot plots showing the gating strategy employed for CD4^+^ T‐cell identification. (b) Histograms and dot plots showing CFSE proliferation assay results gated on CD4^+^ T cells. Histogram percentages represent the ratio of CFSE^low^ cells in CD4^+^‐gated cell populations. Each row of dot plots represents the analyses of cells stimulated with individual peptide or intact capsid antigen using the indicated peptide‐loaded tetramer. The x‐axis represents CFSE staining, with proliferating cells exhibiting a lower intensity of CFSE. Black circles indicate proliferating CD4^+^ T‐cell populations for each peptide. These data are representative of three independent experiments using two animals

To confirm the specificity of the three epitopes, p220 (VP3), p248 (VP1) and p432 (VP4), identified in the TGEM screen, we investigated their ability to induce tetramer‐positive cell proliferation. To do this, PBMCs were first enriched for CD4^+^ T cells by using an antibody cocktail to remove cells expressing CD8, γδ, CD14 or CD21 receptors. As shown in Figure 3, CD4^+^‐enriched PBMC stimulated with each peptide exhibited a matched peptide‐loaded tetramer‐positive population in expanded (CFSE^low^) subsets. In FMD9 PBMC, the frequency of each peptide‐specific CD4^+^ T‐cell population was estimated using these CFSE proliferation assay results as p220 > p432 > p248. We also carried out the CFSE proliferation assay using non‐enriched PBMCs, but only a p220‐specific T‐cell population could be detected (data not shown). Even after FMD7 and FMD9 PBMC underwent CD4^+^ enrichment, an expanded p245‐specific T‐cell population was not observed in CFSE proliferation assays. After conducting three independent cell proliferation assays, p432‐specific proliferation was only detected on one occasion using FMD7 PBMC, while the presence of a p432‐specific T‐cell population was detected on each occasion using FMD9 PBMC.

### The majority of FMDV‐specific CD4^+^ T cells exhibit Th1 phenotype

**FIGURE 4:**
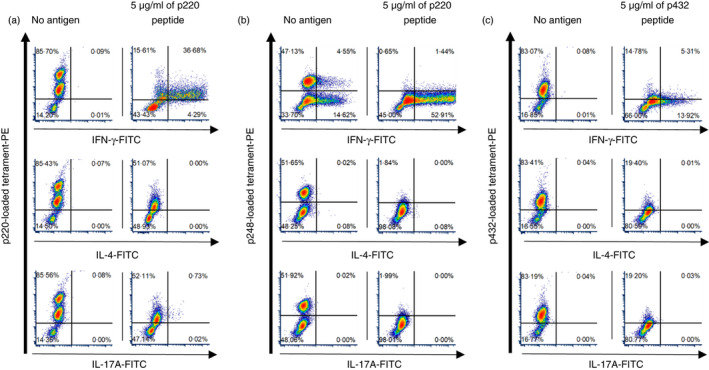
Intracellular cytokine staining of peptide‐expanded CD4^+^ T‐cell‐enriched populations. CD4^+^ T‐cell‐enriched populations were expanded over a 4‐week period and then cultured with autologous PBMCs overnight in the presence or absence of respective peptide. Cells were then treated with brefeldin A for 4 hours to prevent cytokine release prior to staining for the detection of cytokines (IFN‐gamma, IL‐4 and IL‐17A) by flow cytometry. Isotype controls were used to decide the quantile line. Cytokine production by (a) p220‐, (b) p248‐ and (c) p432‐specific CD4^+^ T‐cell populations expanded from FMD9 PBMCs. Cells were gated on lymphocytes, singlets and then CD4^+^ cells. The left plots in each panel show the staining results obtained in the absence of stimulation and the right plots after stimulation with the indicated peptide

To generate adequate levels of peptide‐specific CD4^+^ T cells for further analysis, CD4^+^‐enriched FMD9 PBMCs were expanded over a 4‐week period by two consecutive rounds of antigen stimulation followed by rboIL‐2‐mediated proliferation. The expanded PBMC contained approximately 40‐90% of tetramer^+^ T cells in the CD4^+^ T‐cell population. All expanded peptide (p220, p248, p432)‐specific CD4^+^ T‐cell populations expressed IFN‐γ but not IL‐4 (Figure 4a‐c). These results suggest that DRA/DRB3*020:02 molecule‐restricted epitopes predominantly augment a Th1‐mediated response. Following expansion of both FMD7 and FMD9 PBMC, a minor subpopulation of p220‐specific CD4^+^ T cells was observed that also expressed IL‐17A, a cytokine associated with Th17 subsets (Figure 4a). In comparison with non‐stimulated controls, the intensity of tetramer staining decreased in all cell populations following peptide stimulation (Figure 4a‐c); this was expected as TCR on activated T cells is usually internalized [33, 34].

### FMDV‐specific CD4^+^ T‐cell subpopulations exhibit a memory phenotype *ex vivo*

**FIGURE 5:**
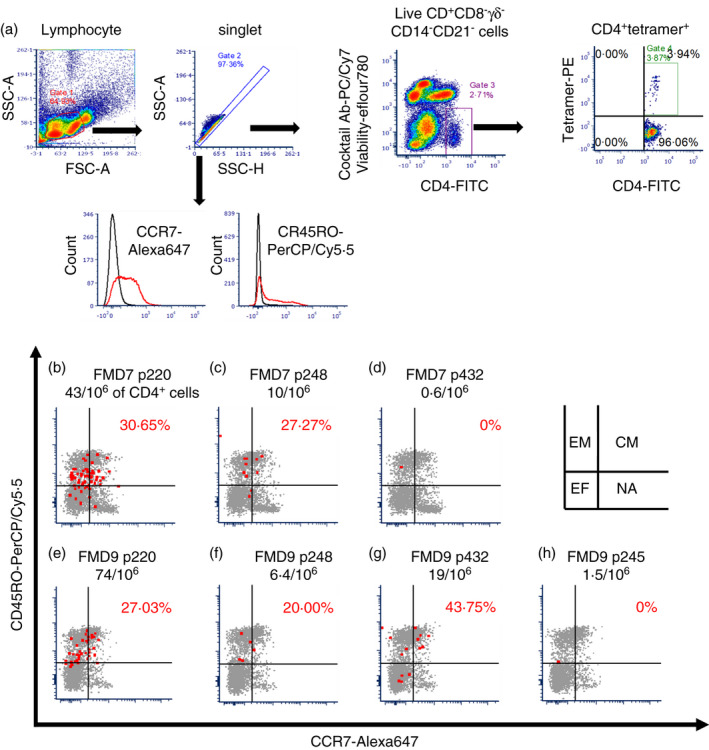
Frequency and phenotype of non‐expanded FMDV epitope‐specific CD4^+^ T‐cell populations *ex vivo*. Non‐expanded PBMCs were stained with loaded tetramer in combination with a cocktail of mAbs for negative selection (CD8, TCR1‐N24 delta chain, CD14 and CD21), mAbs for the detection of CD4, CD45RO and CCR7, and the fixable viability dye eFluor 780. Data were then acquired by flow cytometry. (a) Dot plots, using data obtained with p220‐loaded tetramer–PE, showing the gating strategy for receptor phenotyping of non‐expanded FMDV‐specific CD4^+^ T cells *ex vivo*. (b‐h) The memory phenotype of individual peptide‐specific CD4^+^ T cells is shown for each animal (FMD7 or FMD9) and peptide‐loaded tetramer. Red dots indicate FMDV peptide‐specific CD4^+^ T cells in PE‐enriched PBMC aliquots, and grey dots indicate CD4^+^‐gated cells in pre‐enriched PBMC aliquots. The frequency of peptide‐specific CD4^+^ T cells, per million CD4^+^ T cells in the respective PBMC population, is shown below each animal‐peptide name. The x‐axis represents CCR7‐Alexa 647 and y‐axis CD45RO‐PerCP/Cy5·5 staining. The ratio of CCR7 to CD45RO double‐positive population in peptide‐specific CD4^+^ T cells is shown as a red letter in the upper right quantile. Isotype control antibodies (Rat IgG2aκ antibody–Alexa 647 and mouse IgG3 antibody–PerCP/Cy5·5) were used to decide the quantile line. In each quantile compartment, EM denotes effector memory T cells, CM central memory T cells, EF effector T cells and NA naïve T‐cell populations. These dot plots are representative of two independent experiments using two animals

To further phenotype and enumerate FMDV‐specific CD4^+^ T cells, cryopreserved PBMCs were stained with peptide‐loaded tetramer in combination with mAbs recognizing CD4, memory cell marker CD45RO and CCR7. We determined the *ex vivo* frequency of p220‐, p248‐ and p432‐specific CD4^+^ T cells in approximately 10 million PBMC prepared from FMD7 and FMD9 (Figure 5). To exclude non‐specific stained cells, tetramer^+^ CD4^+^ T cells were obtained with the gating strategy shown in Figure 5a. Peptide p220‐specific CD4^+^ T cells were the most abundant in both FMD7 and FMD9, exhibiting a frequency of 43‐74 per million CD4^+^ T cells (Figure 5b,e). The p248‐specific CD4^+^ T cells were present at a frequency of 6‐10 per million CD4^+^ T cells in both FMD7 and FMD9 (Figure 5c,f). The frequency of p432‐specific CD4^+^ T cells was 19 per million CD4^+^ T cells in FMD9 (Figure 5g); however, none and one p432‐specific CD4^+^ T cells per million CD4^+^ T cells were detected in FMD7 (Figure 5d) in two independent experiments. These results may account for our inability to detect a p432‐specific CD4^+^ T‐cell population by TGEM using FMD7 PBMC. Although p245‐specific CD4^+^ T cells were detected at a frequency of 1‐2 per million CD4^+^ T cells (Figure 5h), it is possible that these results were generated by non‐specific staining due to their very low frequency. The majority of FMD7 and FMD9 peptide‐specific CD4^+^ T cells were CD45RO^+^, but CD45RO^−^CCR7^−^ cell populations were also observed. Effector memory (defined as CD45RO^+^CCR7^−^) cells were the predominant phenotype observed for p220‐ and p248‐specific CD4^+^ T‐cell populations; however, consecutively smaller populations of central memory (defined as CD45RO^+^CCR7^+^) and effector (defined as CD45RO^−^CCR7^−^) cell phenotypes were also detected (Figure 5b,c,e). The p432‐specific CD4^+^ T cells were only confirmed in FMD9, and central memory T cells dominated the population, followed by effector and then effector memory T cells (Figure 5g).

## DISCUSSION

The development of FMD vaccines to date has primarily focused on the characterization of neutralizing and cytotoxic T‐cell epitopes. Until now, such investigations have been hampered by incomplete genome data and a lack of immunological tools that can provide the resolution required to detect small populations of cells. Recent advances in deep sequencing and the application of tetramer‐based methods now offer resources that can be used to better characterize the role bovine memory CD4^+^ T cells play following vaccination. In this study, we have carried out CD4^+^ T‐cell TGEM using bovine PBMCs collected more than four years after vaccination against FMD‐O1/Manisa. Importantly, we have identified three new FMDV/O capsid‐specific CD4^+^ T‐cell epitopes and characterized these memory T cells by phenotyping their receptor and cytokine expression. This work clarifies the presence of long‐lasting helper T cells following FMD vaccination.

The bovine TGEM method allowed us to identify FMDV‐CD4^+^ T‐cell epitopes restricted by the DRA/DRB3*020:02 molecule. To our knowledge, these CD4^+^ T‐cell FMDV epitopes have not been previously reported [9, 10, 11, 12, 13, 14, 15, 16]. Prior to the application of BoLA class II tetramers, it was necessary to either transfect cells with plasmids encoding single MHC class II molecules [35, 36], perform MHC class II‐peptide binding assays [14] or conduct epitope mapping with several MHC homozygous or heterozygous cattle [10] and [11] for identification or estimation of the epitope–MHC class II complex combination. Although the generation of bovine MHC class II tetramer is relatively time‐consuming, tetramer staining was selected over other methods based on its higher specificity and the fact that it facilitates identification of both peptide–MHC complex combinations and peptide‐specific CD4^+^ T cells at the same time.

TGEM and CFSE proliferation assay revealed that the frequency of p245‐specific CD4^+^ T cells in animal FMD9‐ and p432‐specific CD4^+^ T cells in animal FMD7 was extremely low. This was consistent with the results from the tetramer staining of FMDV‐specific CD4^+^ T cells *ex vivo* (Figure 5d,h). In a human study, the detection limit of antigen‐specific CD4^+^ T cells in peripheral blood was one cell per 250,000‐300,000 CD4^+^ T cells *ex vivo* [32, 37]. Because *ex vivo* PBMCs specifically responded to the p432 peptide in one of three independent CFSE proliferation assays, the results of p432‐specific CD4^+^ T cells in FMD7 indicated this is the limit of detection for BoLA class II tetramer staining. On the other hand, p245‐loaded tetramer staining might be non‐specific because the p245 peptide did not stimulate CD4^+^ T‐cell responses in either animal. Taken together, the results (Figure 5c,f) indicate that the detection limit of TGEM from PBMCs was estimated to approximately 6 to 10 antigen‐specific CD4^+^ T cells per million CD4^+^ T cells. In IFN‐γ‐based ELISPOT assays, the typical detection limit of antigen‐specific CD4^+^ T cells is around 50 cells per million PBMCs [38]. In this respect, the TGEM and the BoLA MHC class II tetramer staining are highly sensitive.

Interestingly, none of the epitopes identified in this study were completely exposed to the outer surface region of the FMDV capsid, indicating that they do not overlap with neutralizing antibody epitopes. Amongst the FMDV capsid proteins, VP1 exhibits the highest amino acid sequence diversity and contains the dominant neutralizing epitope within its GH loop [39]. FMDV‐O/UKG/35/2001 peptides, p245 and p248, are in VP1. Peptide p245 (VP1:31‐45) partially overlaps with a previously reported epitope, VP1:35‐49, restricted by the DRB3*002:01, *011:01, *012:01 allele [14], and p248 is located near to p252 (VP1:66‐80), a CD4^+^ T‐cell epitope in animals expressing the DRB3*007:01 allele [10]. This region, VP1:31‐80, may be an abundant recognition site by multi‐BoLA class II molecules. VP3 is a relatively conserved capsid protein in comparison with VP1 and VP2, and there has only been one epitope reported for this capsid region in cattle [14]. The p220 (VP3:116‐130)‐specific CD4^+^ T‐cell population had the highest frequency in both animal FMD7 and FMD9 (Figure 5b,e). Due to their comparatively high frequency and the sequence conservation of this epitope in FMDV serotype O, p220‐specific CD4^+^ T cells appear convenient for investigating memory T‐cell kinetics. In this study, the C‐terminal of the internal VP4 capsid protein was identified as a BoLA‐DRA/DRB3*020:02 molecule‐restricted CD4^+^ T‐cell epitope (p432). The VP4 is highly conserved between FMDV serotypes, making it a potential target to monitor inter‐serotype immunity against FMDV.

With regard to the intracellular cytokine staining, all of the FMDV‐specific CD4^+^ T‐cell populations produced IFN‐γ but not IL‐4, showing that IFN‐γ‐producing Th1 cells were the dominant FMDV‐specific CD4^+^ T cell targeted for epitope presentation by the DRA/DRB3*020:02 molecule. The analysis of IFN‐γ production *in vitro* has showed a positive correlation with vaccine‐induced protection and with reduction in long‐term persistence of FMDV [6, 40]. The frequency of FMDV‐specific Th1 cells could be a key factor of vaccine efficacy against viral persistence and protection. In this study, some of the p248‐specific CD4^+^ T cells maintained IFN‐γ production without fresh addition of specific antigen (Figure 4b). Resting the cells for one week after the preculture may reduce the IFN‐γ response without peptide stimulation. We also observed IL‐17A production by the FMDV/O/p220‐specific CD4^+^ T‐cell subpopulation. IL‐17A is a known proinflammatory cytokine produced in response to infectious pathogens (Ref. [41] IL‐17A treatment was shown to clear the Rhinovirus strain RV1b (*Picornaviridae*, *Rhinovirus*) infection in human epithelial cells, even though this RV1b was able to reduce IL‐17A production by lung CD4^+^ Th17 cells [42]. CD4^+^ T‐cell populations recognizing VP2 epitopes in the capsid of human *Rhinovirus* (RV‐A39 strain) were shown to predominantly comprise of IFN‐γ‐producing cells, but minor subpopulations expressing IL‐17A were also identified [43]. The role of Th17 cells following FMD vaccination remains to be determined, and it may well differ from their role following infection.

The engagement of TCR and peptide‐loaded MHC leads to the formation of an immune synapse and subsequent T‐cell activation initiates TCR internalization [33, 34]. In agreement, we observed a transient reduction in tetramer staining following peptide restimulation of expanded antigen‐specific CD4^+^ T cells and subsequent restoration of staining 48 to 72 hours after stimulation due to TCR replenishment on the cell surface (Figure S3). Our attempts to stain CD4^+^ T cells with tetramer prior to stimulation were unsuccessful (data not shown). In a mouse study, Pastore *et al*., 2019 [44] suggested simultaneous staining with tetramer and cytokine‐specific antibodies after fixation and permeabilization for antigen‐restimulated cells. This procedure may be also useful for our bovine tetramer staining system.

CCR7 is required for cell extravasation through high endothelial venules to enable central memory cells to migrate to secondary lymphoid organs and is used as the first definition for phenotyping human circulating memory T cells into two functionally distinct subsets, central memory and effector memory T cells [45, 46, 47, 48]. In a bovine memory T‐cell study, Vrieling *et al*., 2012 [49] found rat anti‐human CCR7 antibody (clone 3D12) recognized bovine CCR7, and Maggioli *et al*., 2015[50] subsequently defined central or effector memory T cells by CCR7 and CD45RO expression. With regard to the phenotype of FMDV‐specific CD4^+^ T cells identified in this study, most of the observed cells *ex vivo* showed CD45RO expression (Figure 5). Central memory (CD45RO^+^CCR7^+^) cells were observed as a higher frequency FMDV‐specific CD4^+^ T‐cell subpopulation *ex vivo* (Figure 5b,c,e,g), suggesting that circulating central memory cells are required to maintain a long‐lasting CD4^+^ T‐cell response to FMD vaccination. Further studies are needed to determine the number of FMDV‐specific CD4^+^ T cells that correlate with protection from viral challenge.

In summary, we have identified new CD4^+^ T‐cell epitopes in the FMDV capsid and have shown that long‐lasting FMDV‐specific CD4^+^ T cells were present in circulating PBMC more than 4 years after FMD vaccination. Although protection studies were not carried out, we have shown that tetramer‐based analysis can be used to investigate CD4^+^ T‐cell responses to help develop improved vaccines that stimulate enhanced CD4^+^ T‐cell responses and potentially result in enhanced duration of immunity. Future studies will investigate the recognition of FMDV epitopes by other bovine MHC II alleles and will correlate protection from live virus challenge with Th responses induced by vaccination to see whether we can improve correlates of protection based on VNT alone.

## CONFLICTS OF INTEREST

The authors declare that they have no conflicts of interest.

## AUTHOR CONTRIBUTIONS

SM, JN and JS designed the study and acquired funding; SM performed the experiment and wrote the draft of this study; SM, BVC, YH, KM and BC analysed the data; BVC prepared PBMCs from FMDV‐vaccinated animals; JN oversaw BoLA class II tetramer preparation; and JS oversaw the data analysis and prepared manuscripts. All authors contributed to and have approved the final manuscript.

## ETHICAL APPROVAL

All animal experiments were carried out with the approval of the local Ethics Committee at the Pirbright Institute, and in accordance with the Home Office Guidance on the Operation of the Animals (Scientific Procedures) Act 1986 and associated guidelines.

## Supporting information

Fig S1

Fig S2

Fig S3

Table S1
