# Supplementary figures and images for: The detection of long‐lasting memory foot‐and‐mouth disease (FMD) virus serotype O‐specific CD4+ T cells from FMD‐vaccinated cattle by bovine major histocompatibility complex class II tetramer
Source: Immunology. 2021 Jun 8;164(2):266–78. doi: 10.1111/imm.13367 (PMC8442236; doi:10.1111/imm.13367)

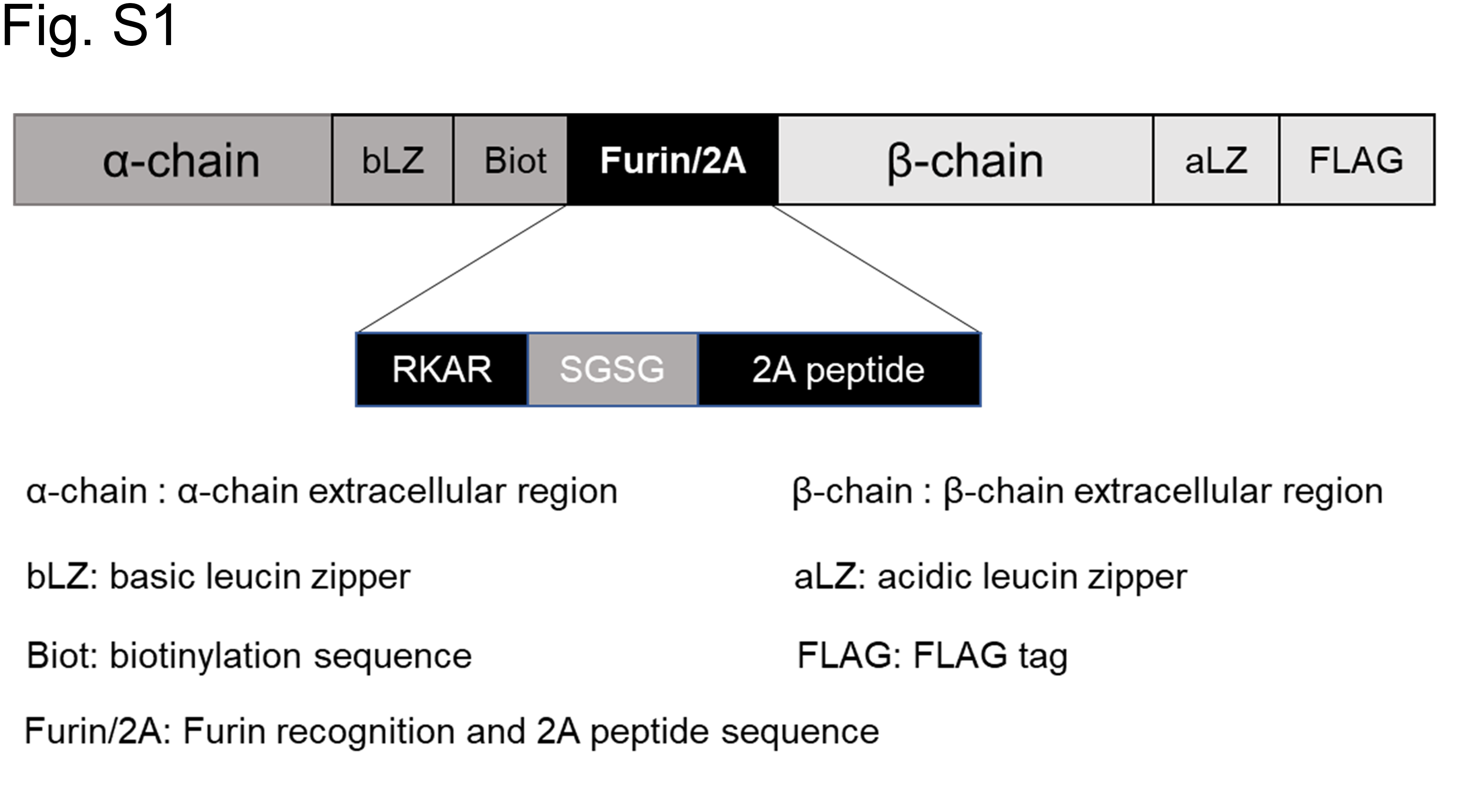

Supplement: Supplementary file 1 — Fig S1 [file IMM-164-266-s004.tif]

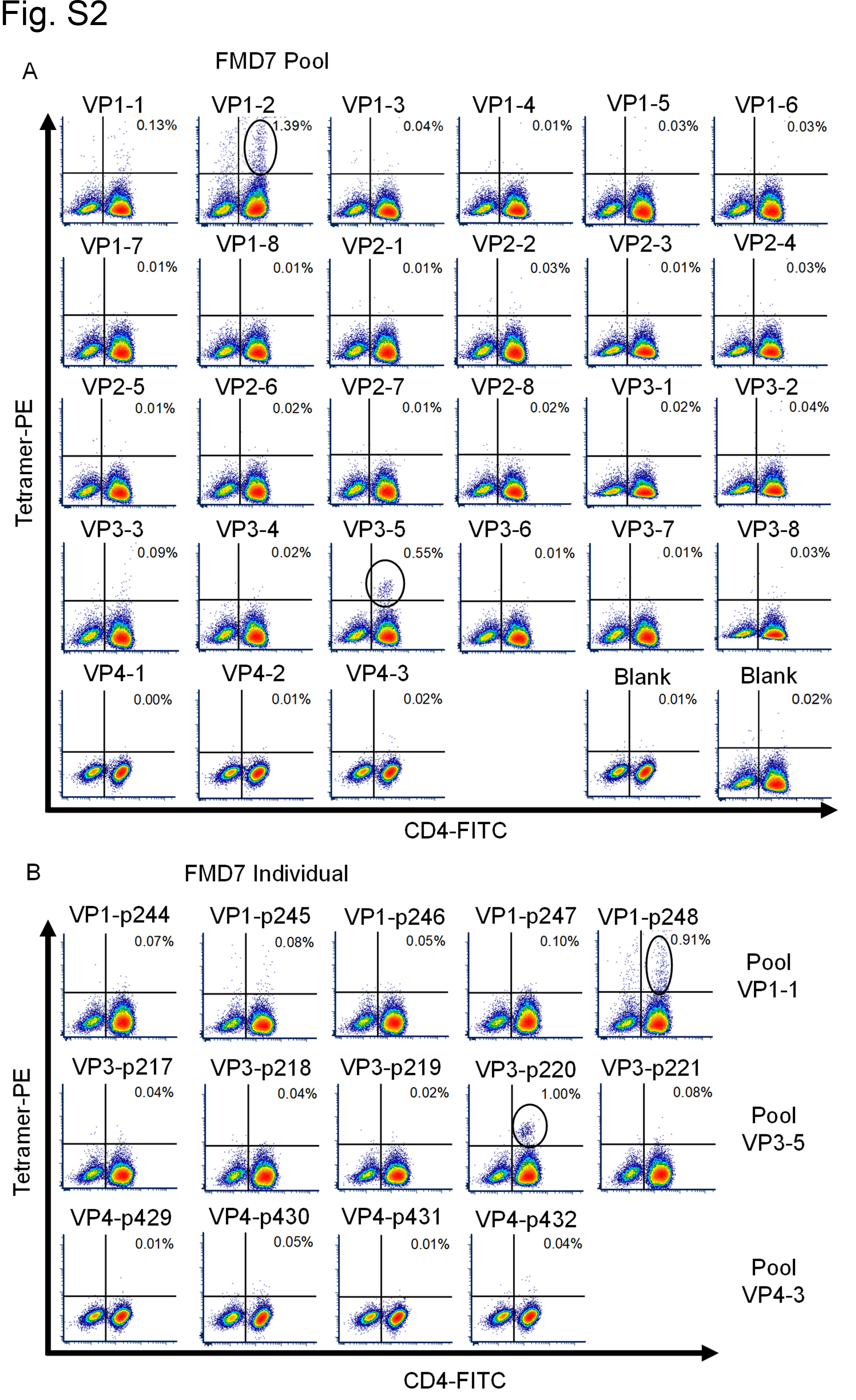

Supplement: Supplementary file 2 — Fig S2 [file IMM-164-266-s002.tif]

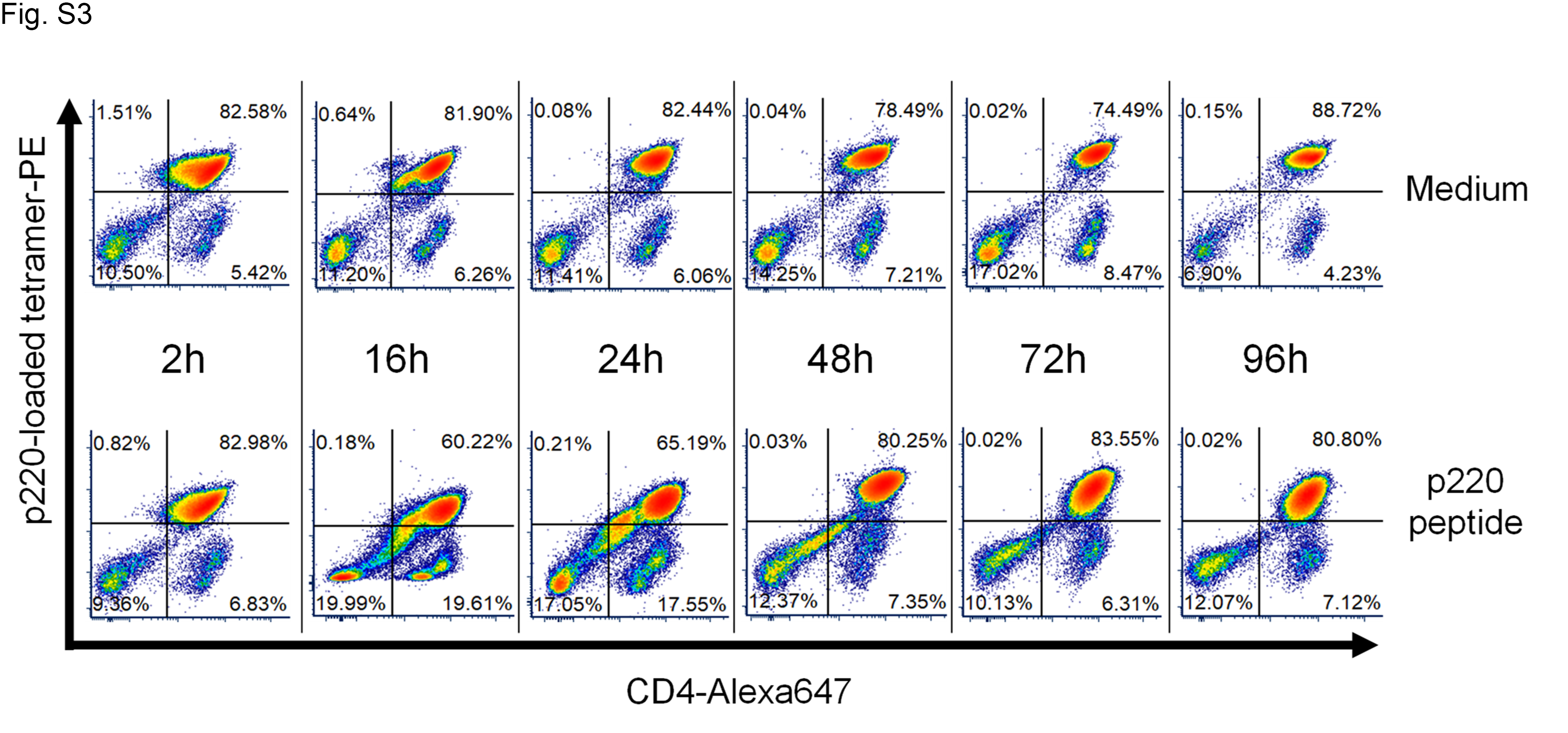

Supplement: Supplementary file 3 — Fig S3 [file IMM-164-266-s001.tif]
