# Supplementary material for: The detection of long‐lasting memory foot‐and‐mouth disease (FMD) virus serotype O‐specific CD4+ T cells from FMD‐vaccinated cattle by bovine major histocompatibility complex class II tetramer
Source: Immunology. 2021 Jun 8;164(2):266–78. doi: 10.1111/imm.13367 (PMC8442236; doi:10.1111/imm.13367)
Supplement: Supplementary file 4 — Table S1 [file IMM-164-266-s003.docx]

Table. S1 Primer set used for BoLA class II tetramer construction

| Primer name | Sequence (5’-3’) |
| --- | --- |
| DRAtet F | AACATGGCCATAACCAGGGTC |
| AfuF2AR1 | GGCTCCGCTTCCGGATCGCTTGGCACGTTCGTGCCATTCGAT |
| AfuF2AR2 | GTCAAAATTCAAAGTCTGCTTTACAGGGGCTCCGCTTCCGGA |
| AfuF2AR3 | CTCGACGTCTCCCGCCAACTTGAGAAGGTCAAAATTCAAAGT |
| AfuF2AR4 | TTTGCCGAATTCGGGCCCAGGGTTGGACTCGACGTCTCCCGC |
| EcoRI-DRB3 F | AAGAATTCGGCATGGTGTGCCTG |
| NotI-FLAG R | AAGCGGCCGCCTACTACTTATCGTCATCG |
| HindIII-DRA F | AAAAGCTTACCATGGCCATAACCAG |
